# Molecular evaluation of the metabolism of estrogenic di(2-ethylhexyl) phthalate in *Mycolicibacterium* sp.

**DOI:** 10.1186/s12934-023-02096-0

**Published:** 2023-04-27

**Authors:** Mousumi Bhattacharyya, Rinita Dhar, Suman Basu, Avijit Das, Darren M. Reynolds, Tapan K. Dutta

**Affiliations:** 1 Department of Microbiology, Bose Institute, EN-80, Sector V, Salt Lake, Kolkata, West Bengal 700091 India; 2 Centre for Research in Biosciences, Department of Applied Sciences, University of the West of England, Bristol, BS16 1QY UK

**Keywords:** Plasticizer, Phthalates, Biodegradation, *Mycolicibacterium*, Estrogenic chemicals, Genome, Transcriptome

## Abstract

**Background:**

Di(2-ethylhexyl) phthalate (DEHP) is a widely detected plasticizer and a priority pollutant of utmost concern for its adverse impact on humans, wildlife and the environment. To eliminate such toxic burden, biological processes are the most promising ways to combat rampant environmental insults under eco-friendly conditions. The present study investigated the biochemical and molecular assessment of the catabolic potential of *Mycolicibacterium* sp. strain MBM in the assimilation of estrogenic DEHP.

**Results:**

A detailed biochemical study revealed an initial hydrolytic pathway of degradation for DEHP followed by the assimilation of hydrolyzed phthalic acid and 2-ethylhexanol to TCA cycle intermediates. Besides the inducible nature of DEHP-catabolic enzymes, strain MBM can efficiently utilize various low- and high-molecular-weight phthalate diesters and can grow under moderately halotolerant conditions. Whole genome sequence analysis exhibited a genome size of 6.2 Mb with a GC content of 66.51% containing 6,878 coding sequences, including multiple genes, annotated as relevant to the catabolism of phthalic acid esters (PAEs). Substantiating the annotated genes through transcriptome assessment followed by RT-qPCR analysis, the possible roles of upregulated genes/gene clusters in the metabolism of DEHP were revealed, reinforcing the biochemical pathway of degradation at the molecular level.

**Conclusions:**

A detailed co-relation of biochemical, genomic, transcriptomic and RT-qPCR analyses highlights the PAE-degrading catabolic machineries in strain MBM. Further, due to functional attributes in the salinity range of both freshwater and seawater, strain MBM may find use as a suitable candidate in the bioremediation of PAEs.

**Supplementary Information:**

The online version contains supplementary material available at 10.1186/s12934-023-02096-0.

## Background

Phthalic acid esters (PAEs) are a class of organic compounds that are primarily dialkyl or alkyl aryl esters of 1,2-benzenedicarboxylic acid, commonly known as plasticizers. Currently, the global production of PAEs has grown from 2.7 million tons in 2007 to over 6 million tons in 2018 while a recent report illustrated the worldwide production of over 11 tons of PAEs per minute [1–3]. The widespread use to increase the durability and flexibility of plastic commercial products including food packaging and processing [4–6] has resulted in ubiquitous distribution of PAEs in the environment. Phthalates are non-covalently bound with polymeric matrices, and thus, can easily leach out into the environment, resulting in their accumulation in sediments and waters [7]. In addition to their detrimental effects on humans as endocrine disrupting chemicals (EDCs) and carcinogens [8–10], PAEs are also toxic to terrestrial and aquatic fauna comprising algae, molluscs, protozoan, crustaceans, invertebrates, and fishes [11]. The toxic phthalates, namely dimethyl phthalate (DMP), diethyl phthalate (DEP), di-*n*-butyl phthalate (D*n*BP), benzyl butyl phthalate (BBP), di(2-ethylhexyl) phthalate (DEHP) and di-*n*-octyl phthalate (D*n*OP) are classified by the United States Environmental Protection Agency (USEPA) as priority pollutants [5, 12]. Amongst the most critical phthalates of environmental concern, DEHP, a high-molecular-weight phthalate, is structurally obstinate and hydrophobic in nature. Nevertheless, due to its advantageous chemical properties, DEHP has been used in a variety of large volume commercial production. For example, polyvinyl chloride tubing contains approximately 40% of DEHP [4]. Due to escalated population growth and rapid industrialization, the European Union (EU) now accounts for one-third of the global production of DEHP, while China accounts for 80% of all PAEs produced [13]. DEHP and its hydrolyzed product mono(2-ethylhexyl) phthalate (MEHP) were found in critically ill neonates and also in adults, causing various anti-androgenic effects leading to reproductive and developmental disorders [14].

Due to the limited availability of appropriate remediation approaches, DEHP contamination remains a significant global problem [15–18]. In this context, microbiological degradation being a cost-effective, efficient, and eco-friendly remediation process, can achieve complete assimilation of environmental pollutants. In the biodegradation of PAEs, several studies unveiled the metabolism of short-chain alkyl phthalates by individual bacteria or bacterial consortia isolated from wastewater, marine, river and sludge sediments [19, 20]. During the last decade, the mounting environmental concern of DEHP had driven the discovery of a number of DEHP-degrading bacterial strains, namely *Acinetobacter* sp. SN13 [21], *Acinetobacter* sp. strain LMB-5 [22], *Microbacterium* sp. J-1 [23], *Rhodococcus spp.* [5, 24–26], *Gordonia* sp. [27], *Pseudoxanthomonas* sp. [28], *Mycobacterium* sp. YC-RL4 [29], *Burkholderia* sp. [30], *Achromobacter* sp. RX [31], *Mycobacterium* sp. DBP42 [32] and *Mycolicibacterium phocaicum* RL-HY01 [33]. Although these studies have primarily revealed biodegradation potential, only a few fully illustrated metabolic pathways at the biochemical level. Moreover, very little is known on the nature of esterase(s) and other catabolic genes involved in assimilating DEHP [34].

In the present study, the *Mycolicibacterium* sp. strain MBM was isolated from plastic contaminated coastal sediment and used to decipher the metabolic pathway of the degradation of DEHP and its hydrolyzed products based on biochemical, genomic, transcriptomic and RT-qPCR analyses, depicting molecular insights on the catabolism of PAEs.

## Results

### Isolation and characterization of microorganism

Enrichment of a plastic waste-contaminated slurry sample in the presence of DEHP facilitated the isolation of a bacterial strain, designated as ‘MBM’. The isolated strain was Gram-positive and revealed nitrate reduction, catalase, tween-80 hydrolysis and urease tests positive, while growth in MacConkey agar, tellurite reduction and gelatin hydrolysis were negative. In addition to above, the Biolog-based phenotypic tests (Additional file 1: Figure S1) indicated that strain MBM belongs to the genus *Mycobacterium*. The complete 16S rRNA sequence of the strain MBM was determined from whole genome sequence analysis and deposited in DDBJ/EMBL/GenBank with the locus tag number L2K20_30630. A BLAST search of the 16S rRNA sequence and comparison to the GenBank nucleotide database revealed 99.34 and 99.21% identity respectively with *Mycolicibacterium neoaurum* strain MN2019 and *Mycolicibacterium neoaurum* strain VKM Ac-1815D, previously known as *Mycobacterium neoaurum* [35] with 100% query coverage. However at the genome level, these strains (GenBank accession numbers, CP006936.2 and CP074376.1) show less than 81% identity with that of strain MBM as determined by the average nucleotide identity calculator (ezbiocloud.net/tools/ani). Phylogenetic relationship of strain MBM with members of well-studied strains of the genus *Mycolicibacterium* in terms of 16S rRNA gene sequence similarity is shown in Additional file 2: Figure S2. Thus, the bacterium was identified as *Mycolicibacterium* sp. strain MBM.

### Growth characteristics

**Fig. 1:**
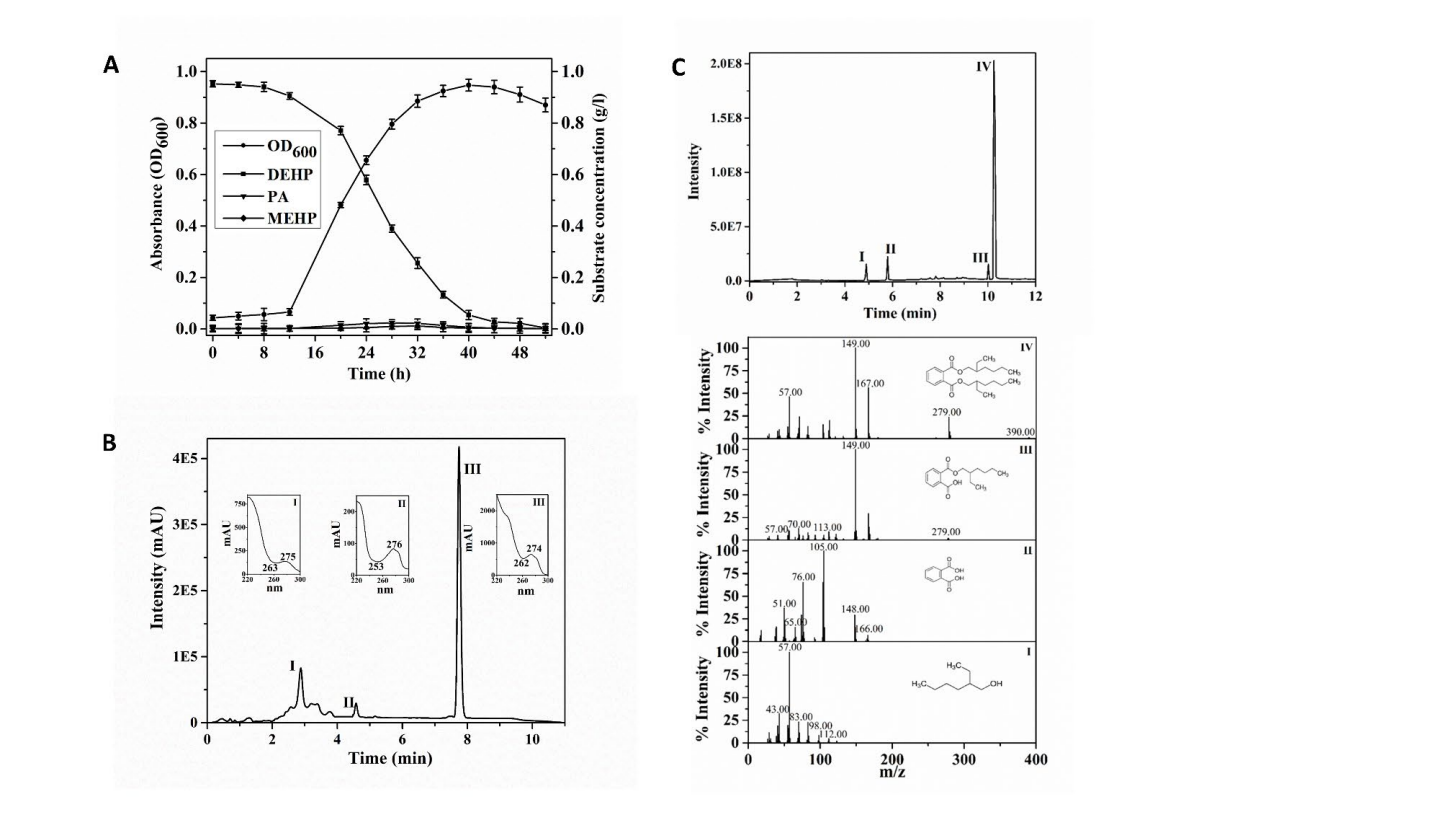
**(A)** Growth of *Mycolicibacterium* sp. strain MBM in mineral salt medium (MSM) upon utilization of di(2-ethylhexyl) phthalate (DEHP) as the sole carbon source under optimal growth conditions in relation to the production of mono(2-ethylhexyl) phthalate (MEHP) and phthalic acid (PA) as transient intermediates. Vertical bars represent mean ± standard deviations from triplicate measurements. **(B)** HPLC profile of di(2-ethylhexyl) phthalate (DEHP) and its metabolic intermediates from the organic extract of the spent culture of strain MBM incubated with DEHP for 28 h. Inset, UV–visible spectra of peaks obtained with diode array analysis. **(C)** GC–MS chromatogram and mass fragmentation patterns for the metabolites of DEHP obtained from the organic extract of the resting cell incubation (30 min) of strain MBM. Peak I, 2-ethylhexanol (2-EH); peak II, phthalic acid (PA); peak III, mono(2-ethylhexyl) phthalate (MEHP) and peak IV, di(2-ethylhexyl) phthalate (DEHP)

Strain MBM was found to be competent in utilizing DEHP as the sole source of carbon and energy. The optimal temperature for the growth of strain MBM in MSM (pH 7.0) was 28 °C with 1.0 g/L of DEHP under shake culture conditions. The growth rate of strain MBM in the presence of DEHP was 0.305 h^− 1^ under optimal growth conditions. Utilization of DEHP by strain MBM was confirmed by its removal from spent culture and a consistent increase in bacterial biomass with incubation time (Fig. 1A). Eventually, DEHP was found to be completely degraded within 52 h. Moreover, the strain MBM can utilize probable pathway intermediates, such as MEHP, phthalic acid (PA), 2-ethylhexanol (2-EH), 2-ethylhexanoic acid (2-EHA) and protocatechuic acid (PCA) individually as sole carbon sources. Interestingly, strain MBM appeared as moderately halotolerant and can utilize DEHP as the sole carbon source under salinity levels up to 60 g L^− 1^ (Additional file 3: Figure S3).

Other than DEHP and its probable metabolic intermediates, the strain MBM was capable of utilizing several other PAEs, including DMP, DEP, D*n*BP, BBP, and D*n*OP (Additional file 4, Table S1). Thus, the strain MBM can more efficiently utilize long-chain dialkyl phthalates in comparison to short-chain dialkyl phthalate and alkyl aryl phthalate. In addition, the strain is also capable of utilizing corresponding hydrolyzed alcohols of the phthalate diesters individually as sole carbon sources. Based on the above, strain MBM appears to encode potential catabolic machineries to completely metabolize many structurally distinct phthalate diesters.

### Respirometric analysis

To understand the oxygen-dependent enzymatic steps, associated with the DEHP degradation pathway in strain MBM, Table 1 summarizes oxygen uptake profiles by strain MBM in the presence of DEHP and various plausible intermediates. Among the tested compounds, PA and PCA are supposed to be metabolized by ring-hydroxylating and ring-cleavage dioxygenases using molecular oxygen as a co-substrate. Conversely, oxygen consumption with 2-EH and 2-ethylhexanal (2-EHALD) indicates the possible involvement of oxidoreductase (NAD^+^-independent alcohol dehydrogenase). Although DEHP and MEHP are metabolized by hydrolytic enzyme(s), oxygen consumption in the presence of these compounds can be explained due to molecular oxygen-dependent metabolism of its hydrolyzed products (PA and 2-EH). All the presented respirometric data were normalized with cell respiration, used as a parallel control.

**Table 1:** Oxygen uptake rates with various substrates by resting cell suspensions of *Mycolicibacterium* sp. strain MBM grown on different compounds^a^

Substrate	Oxygen uptake rate (nmoles of O_2_ consumed /min mg^− 1^ protein) by cells grown on	
	Succinate	DEHP
DEHP	0.0	16.1
MEHP	0.0	21.7
2-Ethylhexanol	0.0	23.1
2-Ethylhexanoic acid	0.0	0.0
2-Ethylhexanal	0.0	20.9
Phthalic acid	0.0	20.4
Protocatechuic acid	0.0	13.9

^a^ All values are corrected for endogenous O_2_ uptake

### Metabolism of DEHP

To understand the metabolic consequences, the organic extract of DEHP-utilizing spent culture was resolved by HPLC analysis and three peaks were observed (Fig. 1B). Based on retention times (RT), UV–visible spectra and co-elution profiles of reference compounds, the three peaks were identified as PA (Peak I, RT 3.03 min), MEHP (peak II, RT 4.53 min) and unutilized DEHP (peak III, RT 7.73 min). However, the hydrolyzed alcohol (2-EH) or its metabolite(s) was not detected by HPLC analysis under the conditions used.

The metabolites of DEHP degradation by strain MBM were also analyzed by GC–MS and the data support the results obtained from the chromatographic analyses. Apart from DEHP [retention time (RT) 10.37 min; *m/z*, 390 (M^+^, 1.2), 279 (23.5), 167 (56.2), 149 (100), 113 (20.0), 104 (15.5), 83 (13.4), 71 (24.2), 57 (46.2)], the pathway intermediates, MEHP [retention time (RT) 10.02 min; *m/z*, 278 (M^+^, 2.1), 167 (29.0), 149 (100), 112 (9.2), 105 (5.2), 93 (5.4), 83 (8.0), 70 (12.7), 57 (9.7)], PA [RT 5.83; *m/z*, 166 (M^+^, 6.8), 149 (15.2 ), 148 (29.1), 105 (100), 104 (65.2), 76 (65.1), 74 (29.1), 65 (15.4)] and 2-EH [RT, 4.85; *m/z*, 112 (4.6), 98 (8.2), 83 (22.5), 71 (11.0), 70 (23.1), 57 (100), 55 (19.4)] were identified from the organic extracts of the MSM-DEHP spent culture (24 h) (Fig. 1C). The presence of MEHP in the MSM-DEHP spent culture, and cell-free extract-DEHP reaction mixture indicates the involvement of esterase/lipase-like enzyme to initiate the DEHP degradation pathway with the release of side-chain alcohol (2-EH). Although no detectable 2-EHALD or 2-EHA was found in both HPLC and GC-MS analyses, these metabolites were presumed to be transient in the degradation process, and 2-EHA was further metabolized via β-oxidation pathway leading to TCA cycle intermediates. Acrylic acid, an inhibitor of β-oxidation pathway, inhibits the 3-ketoacyl-CoA thiolase activity [36, 37] when used at zero hours in the presence of 2-EH or 2-EHA (0.4 mg mL^− 1^) in MSM, no growth of the test organism was detected, which confirmed the involvement of β-oxidation pathway. Furthermore, the metabolism of 2-EH and 2-EHALD by the action of an NAD^+^-independent alcohol/aldehyde dehydrogenase (oxidoreductase) was supported by dye-linked assay for NAD(P)^+^-independent dehydrogenase activity [38, 39] towards 2-EH and 2-EHALD using 2,6-dichlorophenol indophenol (DCPIP) as the ultimate electron acceptor (Additional file 5: Figure S4).

### Enzyme assay

**Fig. 2:**
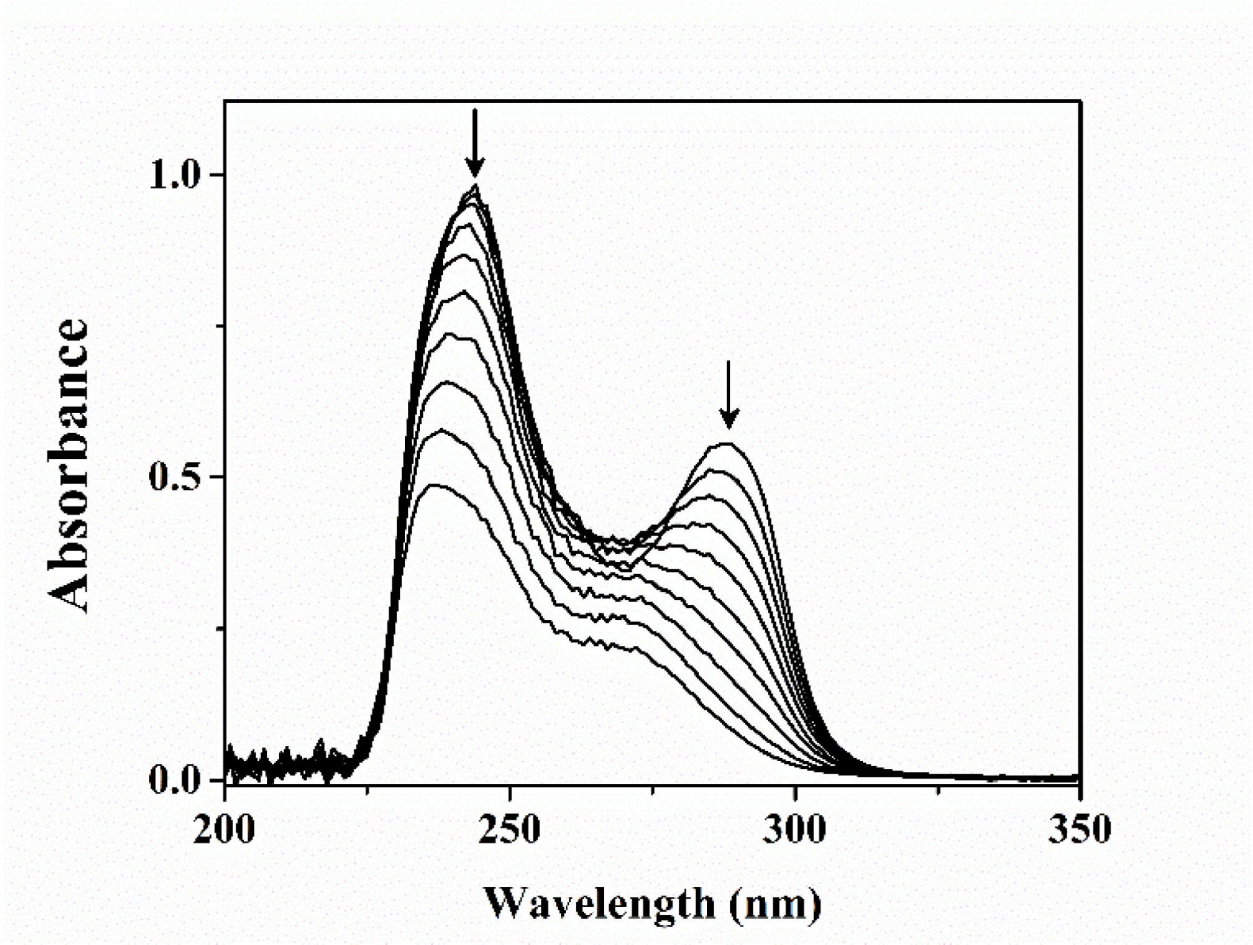
Spectral changes during metabolism of protocatechuic acid (PCA) by the cell-free extract of di-(2-ethylhexyl) phthalate (DEHP)-grown cells of strain MBM. The sample and reference cuvettes contained 50 mM potassium phosphate buffer (pH 7.0) in 1-mL volume. The sample cuvette contained 97 nmol of PCA. Spectra were recorded at 0, 1, 2, 3, 4, 5, 6, 7, 8, 9 and 10 min after the addition 100 µg of crude protein to both cuvettes. Down arrow indicates decreasing absorbance with time

The cell-free extract of a culture of strain MBM grown on DEHP was shown to transform DEHP to MEHP and PA as revealed by TLC analysis, indicating hydrolytic metabolism of the phthalate diester (Additional file 6: Figure S5). Based on the comparison of R_f_ values and optical (UV/fluorescence) properties, MEHP (R_f_ 0.75, light green, fluorescent) and phthalic acid (R_f_ 0.1, black, non-fluorescent) were identified as metabolic intermediates in the metabolism of DEHP (R_f_ 0.94, light green, fluorescent). Nevertheless, the cell-free extracts of strain MBM grown on DEHP or PCA could metabolize PCA in contrast to succinate-grown culture. To determine the participation of *ortho*- or *meta*-cleavage pathway in the metabolism of PCA, the cell-free extracts of culture grown individually in the presence of DEHP and PA were incubated separately with PCA, and both the reaction mixtures remained colorless during incubation. This observation rules out the formation of 2-hydroxy-4-carboxymuconic semialdehyde, the *meta*-cleavage product [40] of PCA, which is yellow in color. Thus, it is assumed that PCA was degraded by *ortho*-cleavage dioxygenase, and this was confirmed by the spectrophotometric determination of the transformation of PCA to β-carboxy-*cis,cis*-muconate by the cell-free extract of strain MBM grown on DEHP or PA (Fig. 2). Thus, a characteristic decrease in the absorption at 290 nm in the optical spectrum indicates the *ortho* cleavage of PCA with the formation of β-carboxy-*cis,cis*-muconate [41–43] where the specific activity was found to be 0.23 µmoles mg^− 1^ protein min^− 1^.

### Genome analysis

**Fig. 3:**
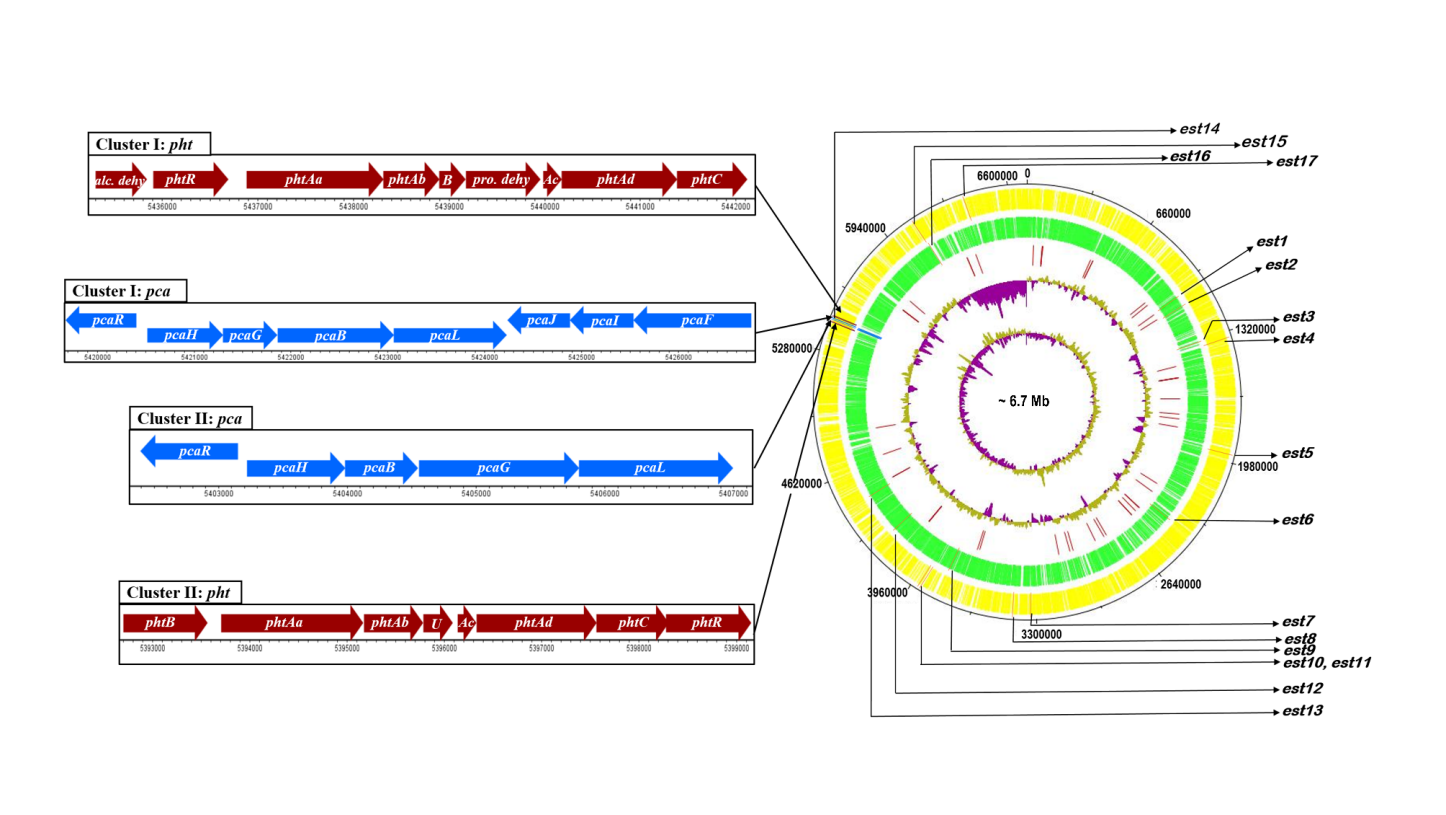
Genome data of *Mycolicibacterium* sp. strain MBM. Circular map of the genome of strain MBM. The tracks from outside represent: (1) Forward CDSs (yellow); (2) Reverse CDSs (green); (3) tRNA (maroon); (4) %GC plot (purple and mustard yellow); (5) GC skew [(GC)/(G + C)] (purple and mustard yellow). The genome of MBM carries two sets of clusters of catabolic genes each for phthalic acid (PA) and protocatechuic acid (PCA) apart from 17 putative phthalate esterase(s) marked as *est1*-*est17*. The genes are drawn to the exact scale. Other genes relevant to phthalate metabolic pathway, besides essential genes for multiple cellular processes are listed in Supplementary file 2 (Supporting Information)

Genome sequencing of strain MBM revealed a genome size of 6,721,756 bp (~ 6.7 Mb) with a GC content of 66.51%. Analysis of the sequence data confirmed the presence of 6,878 coding sequences (CDS), where 1979 were annotated as hypothetical proteins, apart from the occurrence of 150 tRNA genes and 24 rRNA operons. A circular representation of the draft genome highlighting the features of the genome relevant to DEHP degrading genes have been shown (Fig. 3). Initially, in the *de novo* assembly, a total of 332 contigs were constructed. After removal of small contigs (< 500 bp), finally, 242 contigs were obtained. In this analysis, the best kmer size was 127 and the exact N50 value was 194,677 bp while the sequencing coverage was 10×. The identified CDS could be assigned to 50 different categories of clusters of orthologous groups (COGs), suggesting that the organism is efficient in the metabolism of terpenoid, lipids, carbohydrate, amino acid, and a variety of aromatic compounds. (Additional file 7, Table S2). In relation to the esterase-mediated metabolism of phthalate diesters and subsequent catabolism of hydrolyzed side-chain alcohols, the genome of strain MBM harbored 17 putative hydrolases (Additional file 8: Table S3) based on conserved motif/residues as described earlier [44]. Apart from hydrolases, two individual degradative gene clusters each for PA and PCA metabolism were detected (Additional file 8: Table S3, Fig. 3). The genome also harbors a number of genes, annotated as dehydrogenases, oxidoreductases and oxidases for the possible metabolism of hydrolyzed side-chain alcohol, besides several gene clusters encoding for β-oxidation pathway enzymes (Additional file 8: Table S3). Apart from the phthalate assimilating pathway genes, the genome of strain MBM also divulged the the presence of several other prospective catabolic genes for the degradation of various hydrocarbons, including benzoate, a metabolite of benzyl butyl phthalate-hydrolyzed product benzyl alcohol and several essential genes for multiple cellular processes, as revealed by enrichment analysis based on KEGG pathways (Additional file 7: Table S2).

### Transcriptome and RT-qPCR analyses

**Fig. 4:**
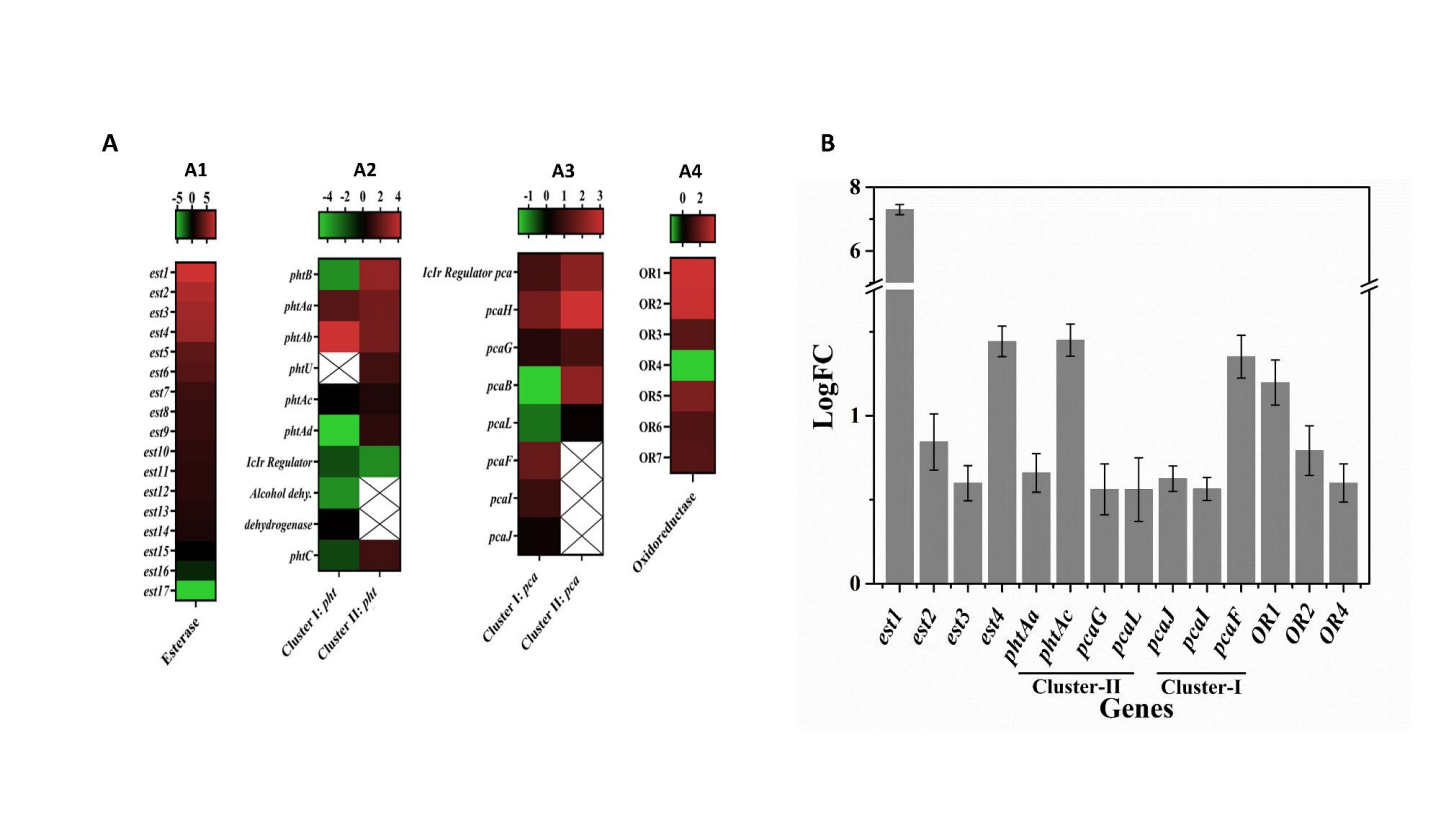
**(A)** The heat map profile of differentially expressed genes, esterase, phthalate metabolic gene (*pht*) cluster I and II. **C** protocatechuate metabolic gene (*pca*) cluster I and II and oxidoreductase. The catabolic gene levels are in the columns and the expression of respective genes is in the rows. Columns are mean-centered, with relative abundance represented by color (green to red from lower to higher abundance), as indicated in the horizontal legend bar. Cross sign indicates absence of gene. **(B)** RT-qPCR analysis of mRNA transcripts of DEHP-degrading catabolic genes obtained from DEHP-grown cells of strain MBM. Relative changes in gene expression were depicted with succinate-grown cells as control, normalized to 16S rRNA. Mean values were obtained from triplicate measurements

Transcriptome analysis revealed a differential regulation of 2359 genes based on comparative analysis of samples obtained from the DEHP-grown culture (MT_2021_00070) with that of succinate-grown culture (MT_2021_00069) of strain MBM using a first cut-off parameter of 0.05 (p-value). Out of the differentially expressed genes, 1140 were upregulated, while the remaining 1219 were downregulated. The top 100 differential loci were used for volcano plot analysis based on most significant p-values (Additional file 9: Figure S6). Out of the probable PAE-degradative pathway genes list, as obtained from the whole genome data (Fig. 3), transcriptomic analysis of DEHP-grown culture funneled down to a relatively smaller number of differentially upregulated genes/gene clusters (Additional file 10: Tables S4). The results of DEHP-induced upregulated genes are represented as a heat map (Fig. 4A) pertaining to respective logFC values.

Using a moderate but reliable logFC cutoff value of 1.5, 11 putative phthalate hydrolases were shown to be upregulated. A phylogenetic analysis of these sequences, along with the representative sequences from 19 known families of esterases, revealed the presence of one mono-esterase (Est3) belonging to the MEHP hydrolase family; two of them were identified as di-monoesterase (Est2 and Est6) belonging to family VIII, while three of them, Est4, Est8 and Est11 were identified as diesterases belonging to family V, IV and VII, respectively [45]. The rest of the phthalate hydrolases (Est1, Est5, Est7, Est9, Est10) did not affiliated with the reported families of esterases (Additional file 11: Figure S7). Among the differentially upregulated esterases, the most probable esterase involved in DEHP hydrolysis is *est1*, with a logFC value of 8.004 followed by *est2*, with a logFC value of 6.789.

Interestingly, out of the two PA-degradative gene clusters, found in the genome of strain MBM (Fig. 3), the cluster II encoding *phtBAaAbUAcAdCR* was found to be upregulated in DEHP-utilizing culture based on transcriptomic analysis. This gene cluster includes a dihydrodiol dehydrogenase (*phtB)*, a multi-component dioxygenase containing large (*phtAa*) and small *(phtAb)* subunits, a ferredoxin (*phtAc*) and a reductase (*phtAd*), apart from a decarboxylase (*phtC*) and an IcIR-type regulator (*phtR*). At the same time, the gene *phtU* of unknown function is also present within the gene cluster, downstream to *phtAb*. Again, the transcriptomic analysis revealed upregulation of the upper operon of the β-ketoadipate pathway of one out of the two available genome-encoded PCA-catabolizing gene clusters in DEHP-utilizing strain MBM. The induced gene cluster *pcaHGBL* (cluster II) encodes a two component ring-cleavage dioxygenase (*pcaHG*), a 3-carboxy-*cis*,*cis*-muconate cycloisomerase (*pcaB*) and a bifunctional (γ-carboxymuconolactone decarboxylase and β-ketoadipate enol-lactone hydrolase) enzyme (*pcaL*). A comparison of dioxygenase subunits *pcaH* and *pcaG* revealed 79.13 and 76.27% sequence identity with the homologous dioxygenase subunits in *Mycobacterium* sp. stain YC-RL4, characterized as PCA *ortho*-cleavage dioxygenase [29]. Thus, the upregulated expression of *pcaHG* corroborates the cell-free extract-mediated enzyme assay revealing PCA *ortho*-cleavage dioxygenase activity (Fig. 2). On the other hand, the lower operon of the β-ketoadipate pathway *pcaFIJ* is encoded in *pca* cluster I (*pcaRHGBLFIJ*) where *pcaIJ* is a two-component β-ketoadipate succinyl-coenzyme A (CoA) transferase and *pcaF* is a β-ketoadipyl-CoA thiolase, transforming β-ketoadipate to succinyl-CoA and acetyl-CoA via β-ketoadipyl-CoA. Transcriptome analysis also revealed an upregulation of five oxidoreductase genes out of the seven differentially expressed oxidoreductase (Fig. 4A), which may be involved in the oxidative transformation of 2-EH to 2-EHA via 2-EHALD. Although transcriptome analysis revealed differential expression of a few annotated β-oxidation pathway genes, the same could not be appropriately assigned to support this multi-enzyme-dependent metabolic pathway.

The selected genes from the transcriptome analysis, which were relevant to the biochemical pathways of degradation of DEHP by strain MBM, were also validated by RT-qPCR experiments (Fig. 4B). The analysis revealed a logFC value of 7.482 for *est1* which correlates well with the transcriptome data. However, *est2, est3* and *est4* showed lower fold-change values, refuting the logFC values as revealed in transcriptome analysis. Correlating with transcriptome data, in contrast to cluster I, both the *pht* and *pca* genes belonging to cluster II showed upregulation in the RT-qPCR study where *phtAa*, *phtAc*, *pcaG* and *pcaL* revealed logFC values of 0.66, 1.45, 0.56 and 0.29, respectively. Upregulation was also observed for the oxidoreductases OR1, OR2 and OR4 with logFC values 1.19, 0.79 and 0.599, respectively, as detected in transcriptome analysis.

### Degradation pathway of DEHP in strain MBM

**Fig. 5:**
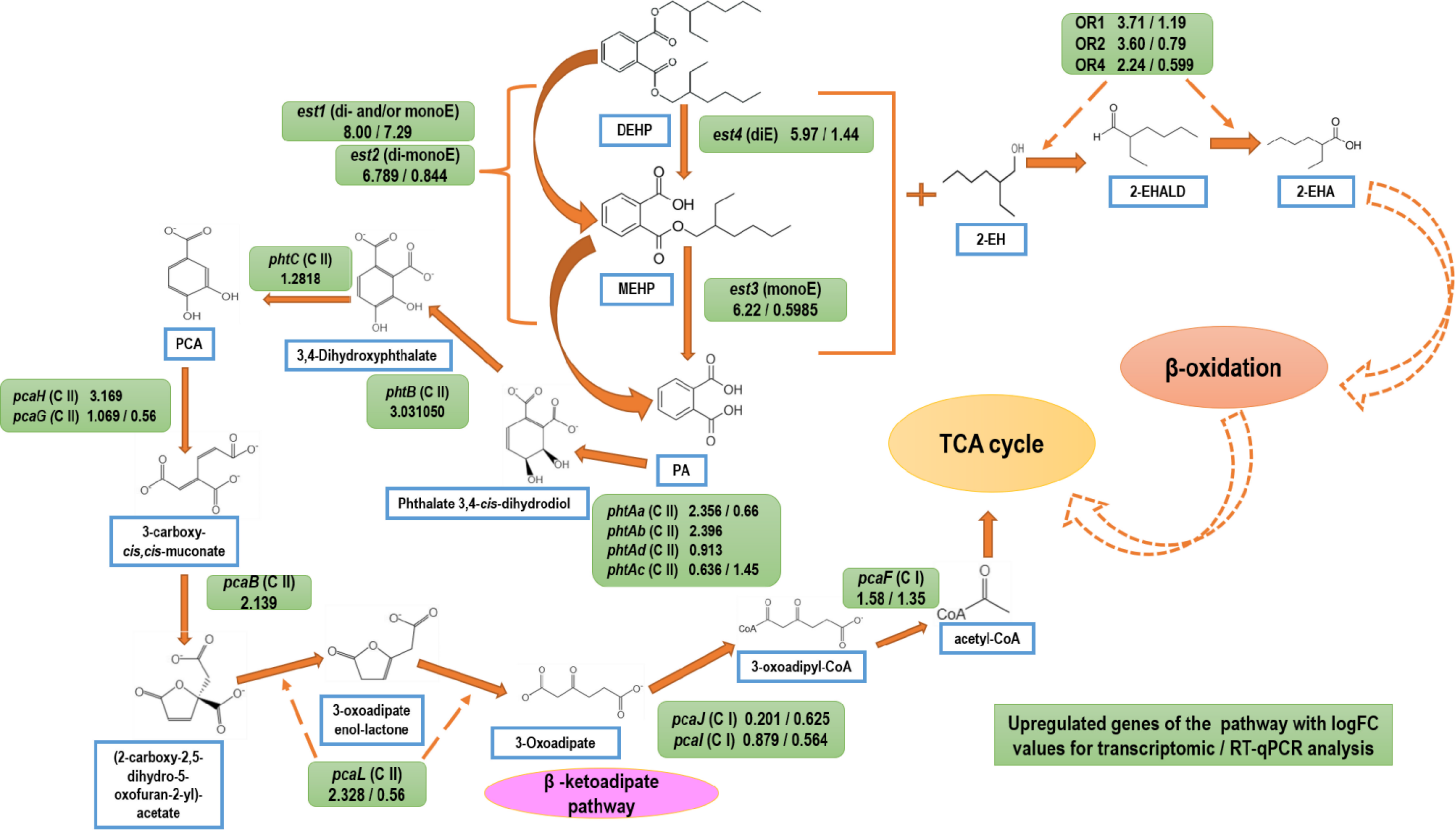
Catabolic pathways evaluated by biochemical, genomic, transcriptomic and RT-qPCR analyses for di(2-ethylhexyl) phthalate (DEHP) degradation in *Mycolicibacterium* sp. strain MBM.

Based on chromatographic, spectrophotometric and enzymatic analyses, the hydrolytic degradation pathway of DEHP was elucidated. One of the hydrolyzed products PA was metabolized via PCA, while the other hydrolyzed product, 2-EH was metabolized by NAD^+^-independent dehydrogenase (oxidoreductase) to 2-EHA via 2-EHALD followed by β-oxidation, ultimately leading to TCA cycle intermediate, suggesting total assimilation of DEHP in strain MBM. In addition, the whole genome sequence analysis of strain MBM revealed genetic reserves essential for the degradation PAEs, while transcriptomic evaluation followed by RT-qPCR analysis further pinned down the catabolic genes founded on DEHP-induced upregulation, reinforcing the suggested metabolic pathway as observed by biochemical studies. The biochemical pathway for the degradation of DEHP in strain MBM depicting the involvement of upregulated genes in various steps of the assimilation process based on genomic/transcriptomic/RT-qPCR analyses is illustrated in Fig. 5.

## Discussion

PAEs were of note given their accelerating use for inducing severe estrogenic effects that lead to the disruption of endocrine systems in humans, wildlife and aquatic species [46, 47]. DEHP, one of the most widely used PAEs, is known to exhibit various toxic effects via the food chain [8, 21]. Microbial remediation offers an eco-friendly and cost-effective approach to restore polluted ecosystem effectively. The DEHP-degrading strain MBM, identified in this study, belongs to a genus *Mycolicibacterium*, which has diverged from the genus *Mycobacterium* belonging to the family *Mycobacteriaceae* [48]. Unlike the genus *Mycobacterium*, where several species are classified as major human pathogens, the genus *Mycolicibacterium* is primarily non-pathogenic, typically found in diverse, non-host-associated environments. Currently, more than 92 recognized species belong to this genus [49] and mostly are saprophytic, capable of processing decaying and decomposing organic matter for nutrients. In general, they can break down hemicellulose and lignin and produce several enzymes including hydrolases, lyases, and esterases to degrade plant cell-wall components, such as chitin, cellulose, pectin, etc. In addition, unlike phytopathogens, they are beneficial in promoting plant growth. In association with plant rhizosphere, *Mycolicibacterium* sp. strains are reported as potent degraders of various polycyclic aromatic hydrocarbons (PAHs) [50–52].

Recently, phthalate remediation was reported in *Mycolicibacterium phocaicum* RL-HY01, a DEHP degrading strain, isolated from an intertidal sediment site [33]. This is the only available report that described a preliminary biochemical study on the degradation of PAE in *Mycolicibacterium* sp., where non-ionic surfactant tween-80 was supplemented for enhanced bacterial growth. Nevertheless, there are a few reports on DEHP degraders, namely *Mycobacterium* sp. NK0301, isolated from soil and sewage sludge, *Mycobacterium* sp. YC-RL4 from petroleum-contaminated site and *Mycobacterium* sp. DBP42 from marine plastics debris [29, 32, 53]. All these studies, primarily revealed biodegradation potential, while only a few illustrated metabolic pathways at the biochemical level. The only report that exploited proteogenomic and metabolomics approaches for DEHP degradation was well documented, but the study fails to confirm the initial enzyme(s) involved in the metabolic process [32].

Due to saprophytic properties, i.e., capable of producing hydrolase/esterase, strain MBM was found to utilize several short- and long-chain dialkyl phthalates and alkyl aryl phthalate, individually as sole carbon sources. Similarly, *Mycolicibacterium* sp. RL-HY01 could also utilize both linear and branched side-chain PAEs, and DEHP was reported to be metabolized via dihexyl phthalate (DHP), D*n*BP, PA, salicylic acid (SA) and gentisic acid (GA), although the enzymatic steps involved in the upper pathway (DEHP to DHP to DBP) remains ambiguous. Unconventional intermediates, such as 2-ethylhexyl pentyl phthalate, butyl (2-ethylhexyl) phthalate (BEHP), mono-hexyl phthalate (MHP) and mono-butyl phthalate (MBP), were reported in DEHP degradation by a halotolerant consortium LF, and again, the proposed degradation pathway could hardly justify enzyme chemistry [46]. Analogous results were reported in *Burkholderia pyrrocinia* B1213 and *Gordonia* sp. Lff [30, 54]. The degradative potential of strain MBM in the metabolism of diverse PAEs is appropriately reflected in the whole genome data, harboring a number of esterases and other relevant catabolic enzymes. Further transcriptome evaluation followed by RT-qPCR analysis allowed us to pin down several DEHP-induced functional enzymes involved in the metabolic process.

Transcriptome data revealed that 11 upregulated esterases are scattered in the genome and are supposed to be under distinct regulation. Of which *est1*, the maximally induced esterase, does not belong to any of the known families of esterases, while the next esterase in the descending order of induction profile is *est2*, affiliated to Group VIII, comprised of β-lactamases and had been described as di-monoesterase, which is supposed to metabolize both phthalate diester as well as phthalate monoester, followed by *est3*, which belongs to Group M and comprises of phthalate monoesterase only.

Transcriptomic data also revealed the upregulation of one of the gene clusters (*phtBAaAbUAcAdCR*) for phthalate metabolism out of the two relevant clusters in the genome. This gene cluster is similar to the phthalate-metabolizing gene cluster reported in *A. keyseri* [55] except for the presence of *phtU*. However, *phtU* in phthalic acid-degrading gene cluster was observed in *Mycolicbacterium vanbaaleni* (previously described as *Mycobacterium vanbaaleni*), *Mycobacterium* sp. DB42 and in *Gordonia* sp. HS-NH1, although the role of *phtU* in PA degradation remains to be defined [32, 56, 57]. The upregulated gene cluster *pcaHBGL* in strain MBM is similar to that found in *Rhodococcus* sp. strain RHA1 and *Rhodococcus opacus* 1CP, where the encoded enzymes can transform PCA to β-ketoadipate [58, 59]. The gene *pcaL*, primarily found in actinobacterial species [59–61], was reported to be a hybrid gene. The expressed protein performs dual function in contrast to that achieved by the expression of *pcaD and pcaC* in the metabolism of γ-carboxy muconolactone to 3-oxoadipate [62]. In addition, the upregulated *pcaFIJ* genes from *pca* gene cluster 1 (Fig. 4A) are possibly involved in the lower pathway of the metabolism of PCA processing 3-oxoadipate to TCA cycle intermediates.

Nevertheless, the RT-qPCR analysis could detect *est1* as the most upregulated esterase apart from both the *pht* and *pca* genes of cluster II, part of the *pca* genes of cluster I, and the oxidoreductase genes (Fig. 4B), as revealed in transcriptome analysis. In some cases, logFC values are less in RT-qPCR in comparison to transcriptome analysis. Often, there are few ‘non-concordant’ genes, which substantially differ in their fold changes in RNA-seq analysis and RT-qPCR evaluation [63]. Among others, various possibilities, such as RNA-seq processing workflows, differences in amplification efficiency, etc., occasionally determine the gene expression levels when quantified with RNA-seq compared to that with RT-qPCR [64, 65].

Generally, the least reported part of PAE degradation is the metabolism of hydrolyzed side-chain alcohol. Most of the available information described the involvement of oxygen-independent NAD^+^-dependent dehydrogense transforming alkanol to alkanoic acid via alkanal and subsequent metabolism via β-oxidation. In the present study, oxygen uptake was recorded in the metabolism of both 2-EH (hydrolyzed product of DEHP) and 2-EHALD, indicating the involvement of enzyme(s) other than NAD^+^-dependent dehydrogenase. To the best of our knowledge, this is the first report of the involvement of NAD^+^-independent dehydrogenase (oxidoreductase) in the metabolism of side-chain alcohol 2-EH and its corresponding aldehyde 2-EHALD in the degradation of DEHP.

## Conclusion

A comprehensive biochemical study using chromatographic, spectrometric, respirometric and enzymatic analyses revealed complete pathway(s) of degradation of DEHP in *Mycolicibacterium* sp. strain MBM. Further genomic, transcriptomic and RT-qPCR analyses illustrated the presence of catabolic machinery and upregulated genes/gene clusters in the metabolism of DEHP in strain MBM. Therefore, the present study highlights the importance in evaluating the catabolic potential of strain MBM in the degradation of PAE and provides a detailed co-relation of biochemical, genomic, transcriptomic and RT-qPCR analyses.

## Methods

### Chemicals

Di(2-ethylhexyl) phthalate (DEHP), mono(2-ethylhexyl) phthalate (MEHP), 2-ethylhexanol (2-EH), 2-ethylhexanoic acid (2-EHA), 2-ethylhexanal (2-EHALD), phthalic acid (PA), protocatechuic acid (PCA), and other phthalate diesters were purchased from Sigma-Aldrich (GmBH, Germany). Methanol, chloroform, and ethyl acetate, both analytical and HPLC grade, were purchased from Merck (India). All other chemicals and reagents used in this study were of analytical grade and used without further purification.

### Bacterial strains and culture conditions

Strain MBM, a DEHP-utilizing bacterium, was isolated from a plastic-contaminated soil slurry sample (Subernarekha - Mohana estuaries, GPS Coordinates: 21° 33’ 44.0244’’ N and 87° 24’ 11.4372’’ E, Digha, West Bengal, India) by enrichment culture technique using DEHP as the sole carbon and energy source. The culture was usually grown in a liquid mineral salt medium (MSM) containing (L^− 1^): 3.34 g *K*_*2*_*HPO*_*4*_, 0.87 g *NaH*_*2*_*PO*_*4*_, 2.0 g *NH*_*4*_*Cl*, 200 mg *MgSO*_*4*_*.7H*_*2*_*O*, 12 mg *FeSO*_*4*_*.7H*_*2*_*O*, 3 mg *MnSO*_*4*_.*H*_*2*_*O*, 3 mg *ZnSO*_*4*_*.7H*_*2*_*O*, and 1 mg *CoCl*_*2*_*.6H*_*2*_*O*; pH adjusted to 7.0 and supplemented with either 1.0 g DEHP, 0.1–1.0 g probable pathway intermediates or 1.0 g of succinate, as sole carbon source. Cultures were incubated on a rotary shaker (180 rpm) at 28 °C. The growth of the cultures was examined by measuring the optical densities at a wavelength of 600 nm in a UV-visible spectrophotometer (Varian, Australia). For resting cell transformation, cells were harvested from late log-phase cultures, suspended in 50 mM potassium phosphate buffer (pH 7.0) and incubated with DEHP or its possible intermediates (0.1-1.0 g L^− 1^) individually as substrate.

### Isolation of metabolites and chemical analysis

Following incubation, spent cultures and resting-cell cultures were extracted thrice with equal volumes of ethyl acetate under acidic conditions (pH 2.0–3.0) obtained using 1.0 N hydrochloric acid. Organic extracts were dehydrated using anhydrous sodium sulfate and evaporated under reduced pressure. Unless stated otherwise, triplicate measurements were performed for all experiments.

To ascertain substrate consumption and accumulation of metabolites, organic extracts of spent cultures were resolved by high-performance liquid chromatography (HPLC), equipped with a Shimadzu LC20-AT pump system, a diode array model SIL-M20A detector and a reversed-phase C18 column connected to a model SIL-20 A autosampler. Metabolites and unconverted substrate were eluted at a flow rate of 1.0 mL min^− 1^ using a gradient solvent system, and the compounds were detected at 254 nm. The mobile phase used to separate unspent substrates and metabolites, comprised of water and methanol, was supplemented with 0.1% (v/v) trifluoroacetic acid. A programmed gradient of the mobile phase included a 3 min linear gradient from 100% (v/v) to 20% (v/v) methanol followed by another 3 min linear gradient from 20% (v/v) to 0% (v/v) methanol and hold at 0% (v/v) methanol for 6 min followed by a ramp from 0% (v/v) to 100% (v/v) methanol in 3 min and hold at 100% (v/v) methanol for 5 min. Identification was established based on comparing retention times and UV-visible spectra of the resolved compounds to those of the authentic compounds analyzed under matching conditions. Quantitative estimation of distinct components was done from the standard curve of the corresponding compounds, constructed by HPLC, operating under identical analytical conditions.

Organic extracts were also used to analyze biodegraded products of DEHP using a PolarisQ mass spectrometer equipped with a Trace GC Ultra chromatograph (Thermo Fischer Scientific Inc., USA) and a DB-624 (30 m × 0.32 mm × 1.8 μm) capillary column (Agilent Technologies, USA). The ion source was kept at 180 °C. The inlet temperature and the transfer line temperature were kept at 240 °C. The column temperature program settings were: a 2 min hold at 70 °C, an increase at 10 °C min^− 1^ to 200 °C, hold for 1 min at 200 °C, an increase at 5 °C min^− 1^ to 240 °C and a hold for 15 min at 240 °C. Helium was used as a carrier gas (1 mL min^− 1^) and an injection sample volume of 1 µL. The electron ionization energy was 70 eV. To identify the metabolites, mass fragmentation patterns were compared with available authentic compounds and from instrumental library searches.

Oxygen (O_2_) uptake by bacterial cells was determined at 25 °C using a polarographic oxygen electrode, Clark-type (YSI model 5331 A oxygen probe), connected to a YSI Model 5300 A biological oxygen monitor (Yellow Springs Instrument Co., Ohio). The reaction mixture contained cell suspension (0.5 mL, 25 mg cells wet weight), substrate (0.5 mL, saturated aqueous solution for both DEHP and MEHP) and 2.5 mL of 50 mM phosphate buffer (pH 7.0). The uptake of dissolved oxygen was monitored for 5 min by supplementing the assay substrate. The aqueous solutions of possible metabolic intermediates of the DEHP degradation pathway were added to give a final concentration of 0.1 mM. The O_2_ uptake rates were corrected for endogenous oxygen consumption and expressed as nmol min^− 1^ mg^− 1^ protein.

### Preparation of cell free extract and enzyme assays

A culture of strain MBM grown on MSM supplemented with DEHP or succinate was centrifuged (8,000×g) for 10 min. The cell pellets were washed twice with potassium phosphate buffer (50 mM, pH 7.0). The cell pellet was then resuspended in the matching buffer (OD_600_ = 1.0), loaded into a pre-cooled French press (One Shot model 182; Constant System Ltd., UK), and was lysed for two cycles at 30,000 lb in ^− 2^ (207 MPa). Cell lysates were centrifuged (12,000×*g*) for 30 min at 4 °C, and the supernatants were used as cell-free enzymes. The Bradford method estimated the protein concentration using bovine serum albumin (BSA) as the standard.

To identify the cell-free extract-mediated transformation of DEHP, organic extracts of the reaction mixture containing cell-free extract and DEHP, incubated for 30 min, were analyzed by thin-layer chromatography (TLC). Silica gel 60 GF254 plate (Merck, Germany) was used as the stationary phase while hexane–chloroform-glacial acetic acid (10:3:2) was employed as the mobile phase, and the chromatogram was visualized at 254 nm under a UV lamp.

Protocatechuate dioxygenase activity in the cell-free enzyme preparation was measured spectrophotometrically [41, 42]. One unit of enzyme activity was defined as the amount of cell-free enzyme preparation which degraded 1 µmol substrate per min. The specific activity was defined as units of enzyme activity per mg of protein. A molar extinction coefficient of 2.3 mM cm^− 1^ for protocatechuic acid (PCA) at 290 nm was used to calculate the enzyme activity [41].

### Nucleic acid extraction and sequencing

For molecular characterization, strain MBM was cultivated in Luria broth (LB) at 28 °C for 24 h, and DNA was extracted using a PureLink Genomic DNA Kit (Invitrogen Thermo Fisher Scientific). The concentration and quality of extracted DNA were determined using a Multimode Microplate Reader (PHERAstar® FSX, BMG LABTECH, Germany). The genomic DNA was sequenced using the Illumina MiSeq platform with paired-end technology (2 × 250 bp) according to the manufacturer’s instruction (Illumina, Inc, USA). Nextera XT DNA Library Preparation Kit from Illumina was used to prepare sequencing library. FastQC_v0.115 tool (https://www.bioinformatics.babraham.ac.uk/projects/fastqc) was used separately to analyze both paired-end sequencing read files, and necessary trimming was achieved by FASTX Toolkit v0.0.14 (http://hannonlab.cshl.edu/fastx_toolkit/). The estimation of the best suitable kmer size in the processed reads of the genome of strain MBM was made by employing kmergenie_v1.7051 software with auto Kmer mode as the default parameter [66]. In the *de novo* assembly, good-quality processed reads were subjected to SPAdes_v3.15.0 assembler [67]. For mismatch correction, the analysis was performed by employing the “careful” option. The obtained contigs were aligned using Mauve for genome alignment [68] using the closest match genome of *Mycolicibacterium holsaticum* as a reference. Functional annotation of this genome was done using RAST [69]. The circular map was generated using DNAPlotter [70]. Default parameters were used for all software applications, unless otherwise stated.

### RNA isolation, transcriptome, and RT-qPCR analyses

To isolate total RNA, mid-exponential-phase cultures of strain MBM, grown on DEHP or succinate as the sole carbon source, was subjected to TRIzol reagent treatment according to the manufacturer’s instructions (Invitrogen, USA) following the method described earlier [71] RNase-free DNase I (Thermo Scientific, USA) treatment was done for 1 h at 37 °C to remove DNA contaminants present in the RNA samples, followed by inactivation of the enzyme with EDTA at 70 °C for 10 min. Total RNA was quantified spectrophotometrically at 260 nm, and purity was estimated from the 260/280 absorbance ratio. The integrity of purified RNA was checked by agarose (2%) gel electrophoresis, and an RNA sequencing (RNA-seq) experiment was carried out using the Illumina MiSeq platform. RNA-seq analysis was performed following a pipeline Fastqc-STAR-htseq-count-edgeR. Quality control, filtering, and trimming of RNA read were done by using cut adapt tool [72]. Read mapping of RNA-seq data was done by the STAR aligner (v.2.5.4b) [73] using the genome of strain MBM as a reference. Further, the differential expression analysis was done with the help of R-package edgeR (v.3.32.1) [74]. For gene ontology (GO) enrichment analysis, a web tool ShinyGO was utilized (http://bioinformatics.sdstate.edu/go/).

Quantitative PCR (qPCR) was done in 96-well qPCR plates using ABI 7500 Fast detection system (Applied Biosystems, USA). PCR reactions were carried out using 0.5 µL of gene-specific cDNA samples and that of 16S rRNA as templates with 1 mM final concentration of each of the forward and reverse RT primers (Additional file 12: Table S5) and 4 µL of 2× SYBR green PCR mix in a final volume of 10 µL. The PCR program included a 10 min denaturation step at 95 °C, followed by 40 cycles of 15 s of denaturation at 95 °C and 30 s of primer hybridization at 52 °C and 30 s of polymerization at 60 °C. Relative gene expression levels were calculated using the comparative threshold amplification cycle (CT) by 2^−∆∆CT^ method [75]. The 16S rRNA gene was kept as an endogenous control for each set of reactions, whereas cDNAs of uninduced cells were taken as reference samples.

## Electronic supplementary material

Below is the link to the electronic supplementary material.

**Additional file 1: Figure S1 **
Biolog GEN III MicroPlate plate analysis depicting carbon source utilization under aerobic growth conditions of strain MBM.

**Additional file 2: Figure S2 **
Phylogenetic tree based on 16S rRNA gene sequence of the isolate MBM and other related species from the NCBI database. Accession numbers of the sequences used in this study are shown in parentheses after the strain designation. The tree was rooted using *Nocardia asteroids* as the outgroup sequence. Numbers at nodes are percentage bootstrap values based on 1,000 replications; only values greater than 50% are shown. Bar 0.001 substitutions per nucleotide position.

**Additional file 3: Figure S3 **
Growth of strain MBM in MSM in presence of di(2-ethylhexyl) phthalate (1 g L^− 1^) under different NaCl concentration.

**Additional file 4: Table S1 **
Substrate utilization profile for *Mycolicibacterium* sp. strain MBM.

**Additional file 5: Figure S4 **
Microtiter-plate-based activity assay using 2,6-dichlorophenol indophenol (DCPIP) with strain MBM. Each assay well contained 20 mM phenazine methosulfate, 6.7 mM DCPIP and 50 mM phosphate buffer (pH 7.0). Lane A, absence of substrate and enzyme (negative control); Lane B, addition of 2-ethylhexanol (2-EH) in the first well and 2-ethylhexanal (2-EHALD) in the second well (substrate control), Lane C, additional presence of succinate-induced cell-free extract (CFE) of strain MBM in wells as stated in Lane B while third well contains CFE only, without any substrate (2-EH or 2-EHALD); Lane D, supplementation of DEHP-induced cell-free extract (CFE) of strain MBM in wells as stated in Lane B while third well contains DEHP-induced CFE only, without any substrate (2-EH or 2-EHALD). The assay plate was incubated for 24 h.

**Additional file 6: Figure S5 **
TLC profile of cell-free extract-mediated transformation of di(2-ethylhexyl) phthalate (DEHP) on silica gel GF_254_ plate. Lane 1, Organic extract of cell-free extract-catalyzed reaction mixture, incubated for 30 min; lane 2–5, authentic DEHP, mono(2-ethylhexyl) phthalate (MEHP), phthalic acid (PA) and protocatechuic acid (PCA), respectively.

**Additional file 7: Table S2 **
Enrichment analysis of prospective catabolic genes from genome data of strain MBM based on KEGG pathways.

**Additional file 8: Table S3 **
Probable DEHP degradation pathway genes present in the genome of strain MBM.

**Additional file 9: Figure S6 **
Volcano plot prepared using the R-library Enhanced Volcano, to represent the differentially expressed genes at p-value cutoff below 0.05, and logFC cutoff value of 0. All the genes marked in red are differentially expressed, while the ones marked in green are not.

**Additional file 10: Table S4 **
Differentially expressed DEHP-catabolic pathway genes in strain MBM.

**Additional file 11: Figure S7 **
Phylogenetic analysis of differentially upregulated phthalate hydrolases from strain MBM grown in DEHP-MSM. The upregulated hydrolases are shown in bold letters, while all the enzymes in the tree are mentioned along with respective GenBank accession number and organism name. Numbers at the nodes indicate the levels of bootstrap support based on neighbor joining analysis of 100 resampled data sets. Bootstrap values below 50% are not shown. The scale bar represents 0.10 substitutions per nucleotide position. Multiple sequence alignment was performed using Clustalx v.2 with the inclusion of representative protein sequences from 19 different esterase families. The phylogenetic tree was constructed using neighbour joining algorithm as implemented in Tree ViewX explorer to understand the phylogenetic affiliation of all the upregulated phthalate hydrolases. Halido hydrolase from *Xanthobacter autotrophicus* was used as an outgroup sequence.

**Additional file 12: Table S5 **
Primers used for RT-qPCR analysis of selected genes obtained from genome sequence of strain MBM to evaluate genes involved in the degradation of DEHP.

## Data Availability

The Whole Genome Shotgun project with respect to *Mycolicibacterium* sp. strain MBM was deposited in DDBJ/ENA/GenBank under the accession number JAKJHY000000000. The transcriptome data were deposited in BioProject in GenBank via Bioproject number PRJNA798576 accessible at: https://www.ncbi.nlm.nih.gov/bioproject/PRJN.

## Abbreviations

DMP: dimethyl phthalate
DEP: diethyl phthalate
D*n*BP: di-*n*-butyl phthalate
BBP: benzyl butyl phthalate
DEHP: di(2-ethylhexyl) phthalate
D*n*OP: di-*n*-octyl phthalate
MEHP: mono(2-ethylhexyl) phthalate
PA: phthalic acid
PCA: protocatechuic acid
2-EH: 2-ethylhexanol
2-EHALD: 2-ethylhexanal
2-EHA: 2-ethylhexanoic acid
