# Supplementary material for: Molecular evaluation of the metabolism of estrogenic di(2-ethylhexyl) phthalate in Mycolicibacterium sp
Source: Microb Cell Fact. 2023 Apr 27;22:82. doi: 10.1186/s12934-023-02096-0 (PMC10134610; doi:10.1186/s12934-023-02096-0)
Supplement: Supplementary file 1 — Additional file 1: Figure S1 Biolog GEN III MicroPlate plate analysis depicting carbon source utilization under aerobic growth conditions of strain MBM. [file 12934_2023_2096_MOESM1_ESM.pptx]

## Slide 1
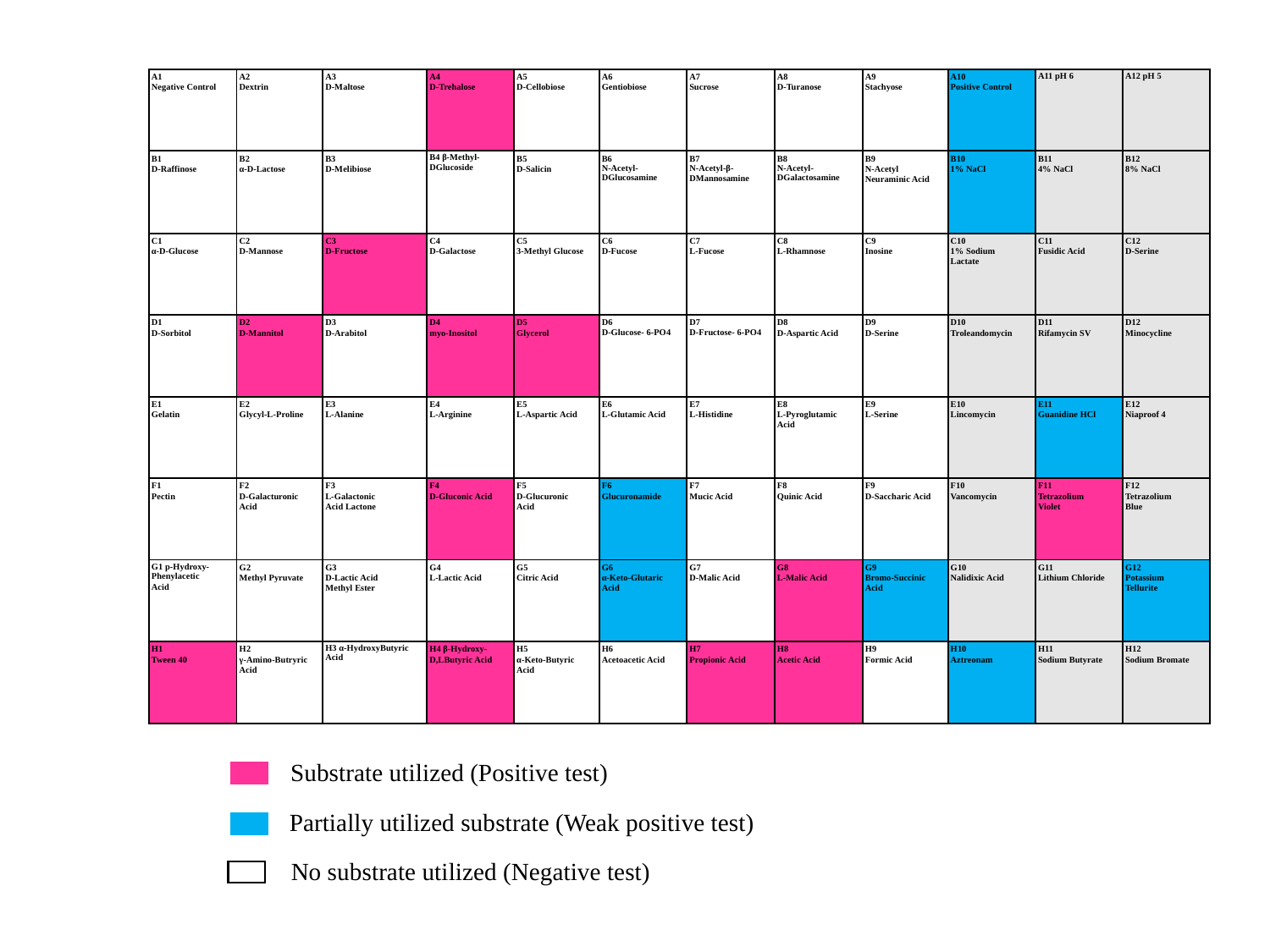

| A1 Negative Control | A2 Dextrin | A3 D-Maltose | A4 D-Trehalose | A5 D-Cellobiose | A6 Gentiobiose | A7 Sucrose | A8 D-Turanose | A9 Stachyose | A10 Positive Control | A11 pH 6 | A12 pH 5 |
| --- | --- | --- | --- | --- | --- | --- | --- | --- | --- | --- | --- |
| B1 D-Raffinose | B2 α-D-Lactose | B3 D-Melibiose | B4 β-Methyl-DGlucoside | B5 D-Salicin | B6 N-Acetyl-DGlucosamine | B7 N-Acetyl-β-DMannosamine | B8 N-Acetyl-DGalactosamine | B9 N-Acetyl Neuraminic Acid | B10 1% NaCl | B11 4% NaCl | B12 8% NaCl |
| C1 α-D-Glucose | C2 D-Mannose | C3 D-Fructose | C4 D-Galactose | C5 3-Methyl Glucose | C6 D-Fucose | C7 L-Fucose | C8 L-Rhamnose | C9 Inosine | C10 1% Sodium Lactate | C11 Fusidic Acid | C12 D-Serine |
| D1 D-Sorbitol | D2 D-Mannitol | D3 D-Arabitol | D4 myo-Inositol | D5 Glycerol | D6 D-Glucose- 6-PO4 | D7 D-Fructose- 6-PO4 | D8 D-Aspartic Acid | D9 D-Serine | D10 Troleandomycin | D11 Rifamycin SV | D12 Minocycline |
| E1 Gelatin | E2 Glycyl-L-Proline | E3 L-Alanine | E4 L-Arginine | E5 L-Aspartic Acid | E6 L-Glutamic Acid | E7 L-Histidine | E8 L-Pyroglutamic Acid | E9 L-Serine | E10 Lincomycin | E11 Guanidine HCl | E12 Niaproof 4 |
| F1 Pectin | F2 D-Galacturonic Acid | F3 L-Galactonic Acid Lactone | F4 D-Gluconic Acid | F5 D-Glucuronic Acid | F6 Glucuronamide | F7 Mucic Acid | F8 Quinic Acid | F9 D-Saccharic Acid | F10 Vancomycin | F11 Tetrazolium Violet | F12 Tetrazolium Blue |
| G1 p-Hydroxy- Phenylacetic Acid | G2 Methyl Pyruvate | G3 D-Lactic Acid Methyl Ester | G4 L-Lactic Acid | G5 Citric Acid | G6 α-Keto-Glutaric Acid | G7 D-Malic Acid | G8 L-Malic Acid | G9 Bromo-Succinic Acid | G10 Nalidixic Acid | G11 Lithium Chloride | G12 Potassium Tellurite |
| H1 Tween 40 | H2 γ-Amino-Butryric Acid | H3 α-HydroxyButyric Acid | H4 β-Hydroxy-D,LButyric Acid | H5 α-Keto-Butyric Acid | H6 Acetoacetic Acid | H7 Propionic Acid | H8 Acetic Acid | H9 Formic Acid | H10 Aztreonam | H11 Sodium Butyrate | H12 Sodium Bromate |
Substrate utilized (Positive test)
Partially utilized substrate (Weak positive test)
No substrate utilized (Negative test)
