# Supplementary figures and images for: Molecular evaluation of the metabolism of estrogenic di(2-ethylhexyl) phthalate in Mycolicibacterium sp
Source: Microb Cell Fact. 2023 Apr 27;22:82. doi: 10.1186/s12934-023-02096-0 (PMC10134610; doi:10.1186/s12934-023-02096-0)

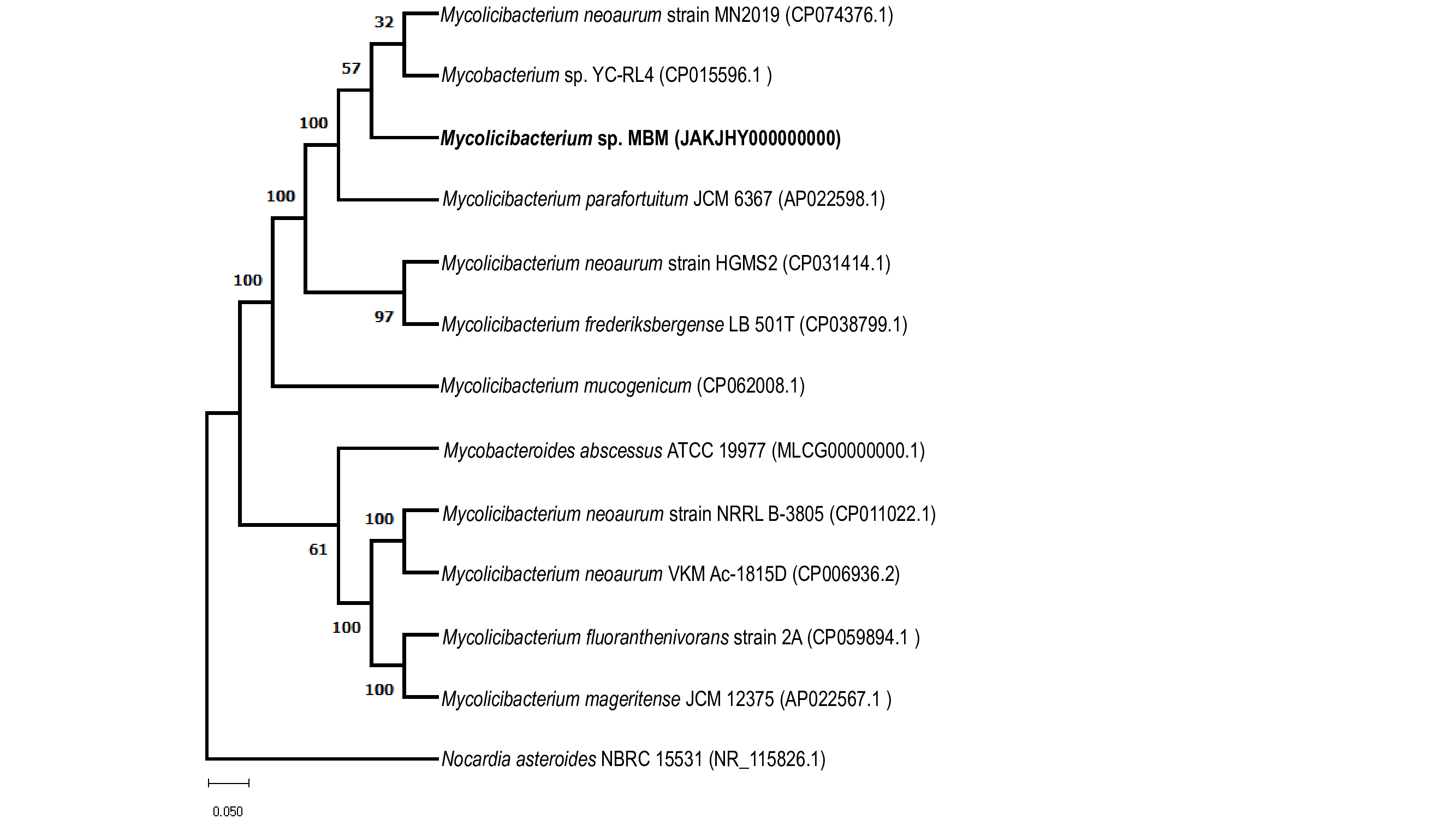

Supplement: Supplementary file 2 — Additional file 2: Figure S2 Phylogenetic tree based on 16S rRNA gene sequence of the isolate MBM and other related species from the NCBI database. Accession numbers of the sequences used in this study are shown in parentheses after the strain designation. The tree was rooted using Nocardia asteroids as the outgroup sequence. Numbers at nodes are percentage bootstrap values based on 1,000 replications; only values greater than 50% are shown. Bar 0.001 substitutions per nucleotide position. [file 12934_2023_2096_MOESM2_ESM.tiff]

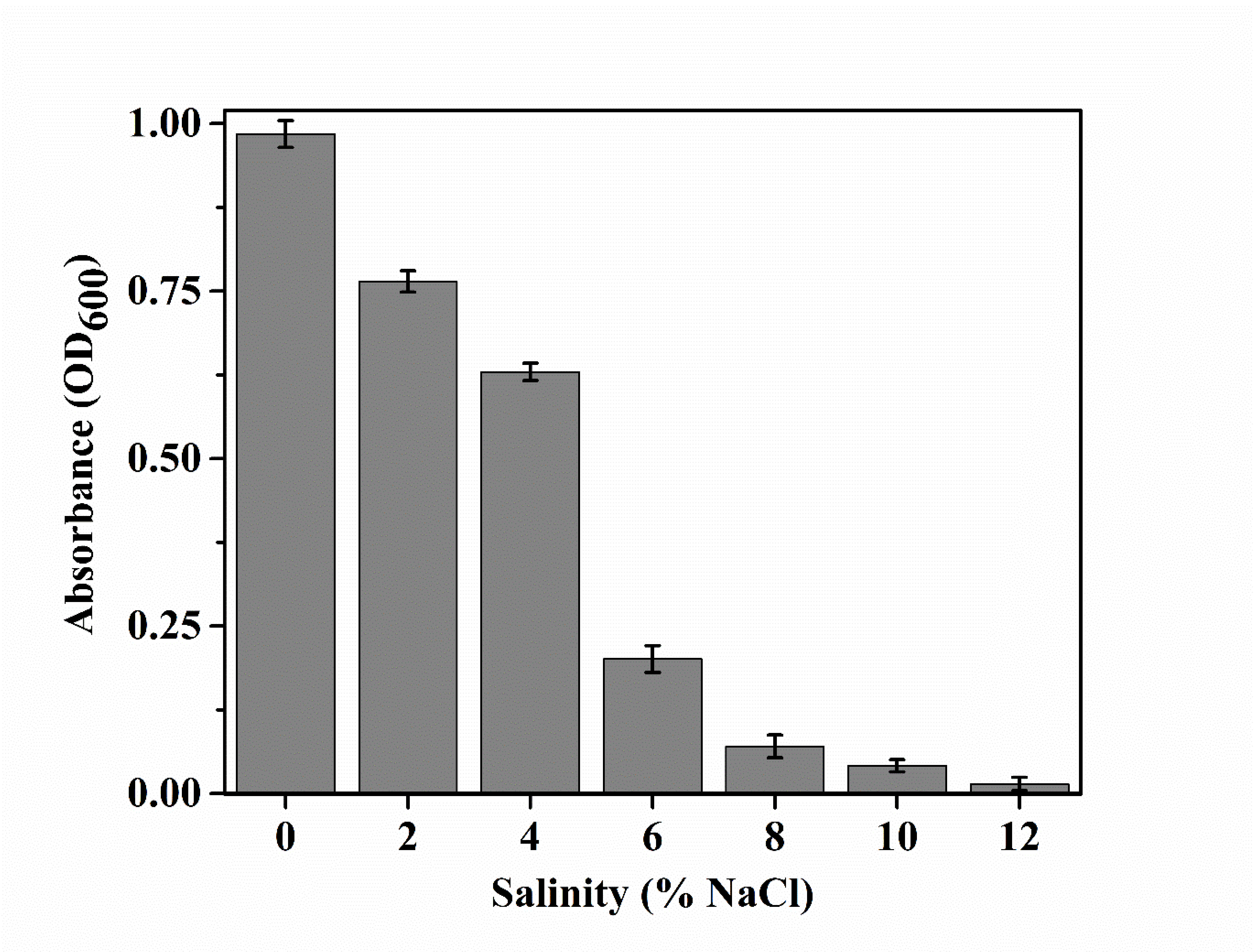

Supplement: Supplementary file 3 — Additional file 3: Figure S3 Growth of strain MBM in MSM in presence of di(2-ethylhexyl) phthalate (1 g L− 1) under different NaCl concentration. [file 12934_2023_2096_MOESM3_ESM.tif]

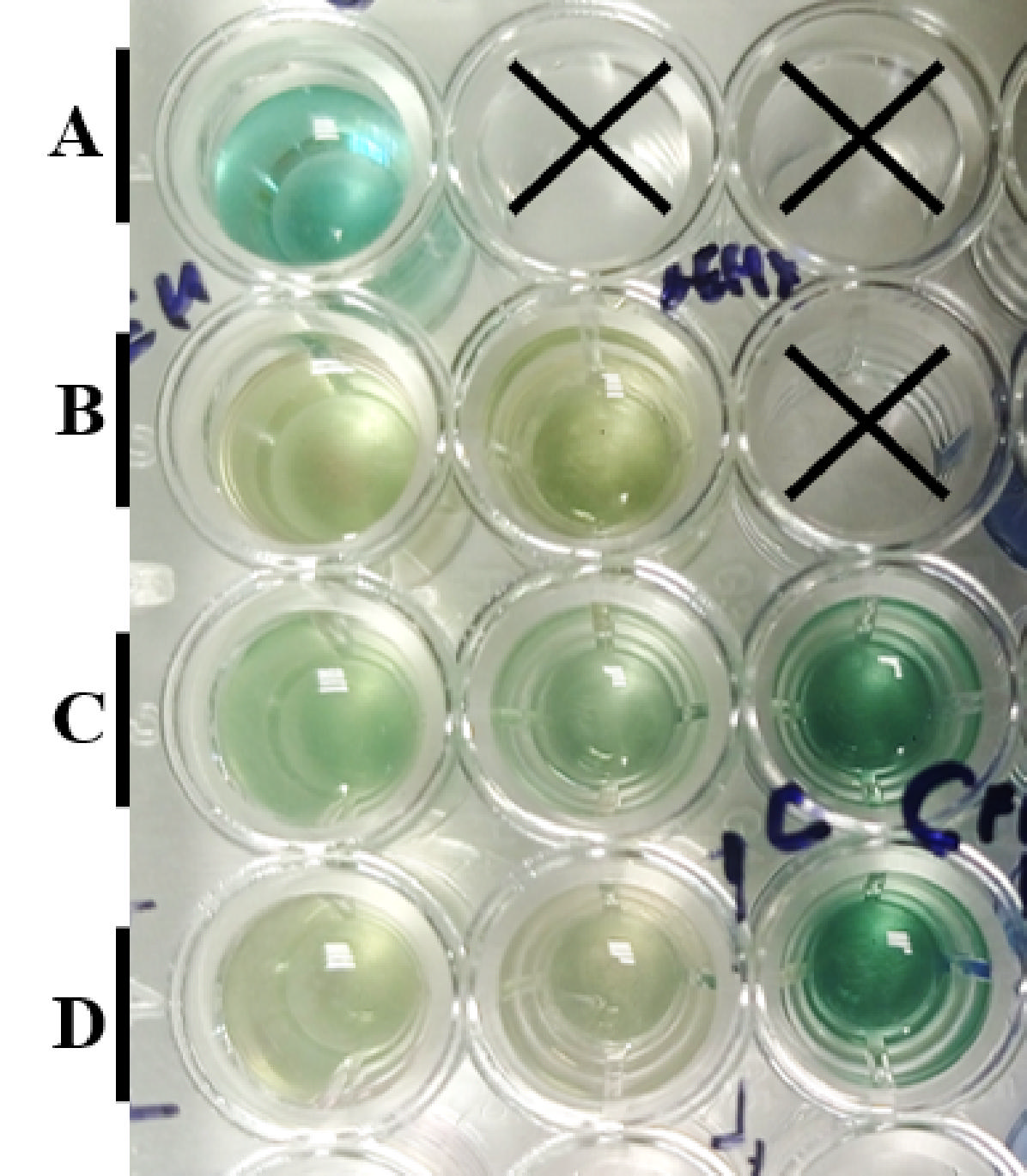

Supplement: Supplementary file 5 — Additional file 5: Figure S4 Microtiter-plate-based activity assay using 2,6-dichlorophenol indophenol (DCPIP) with strain MBM. Each assay well contained 20 mM phenazine methosulfate, 6.7 mM DCPIP and 50 mM phosphate buffer (pH 7.0). Lane A, absence of substrate and enzyme (negative control); Lane B, addition of 2-ethylhexanol (2-EH) in the first well and 2-ethylhexanal (2-EHALD) in the second well (substrate control), Lane C, additional presence of succinate-induced cell-free extract (CFE) of strain MBM in wells as stated in Lane B while third well contains CFE only, without any substrate (2-EH or 2-EHALD); Lane D, supplementation of DEHP-induced cell-free extract (CFE) of strain MBM in wells as stated in Lane B while third well contains DEHP-induced CFE only, without any substrate (2-EH or 2-EHALD). The assay plate was incubated for 24 h. [file 12934_2023_2096_MOESM5_ESM.tif]

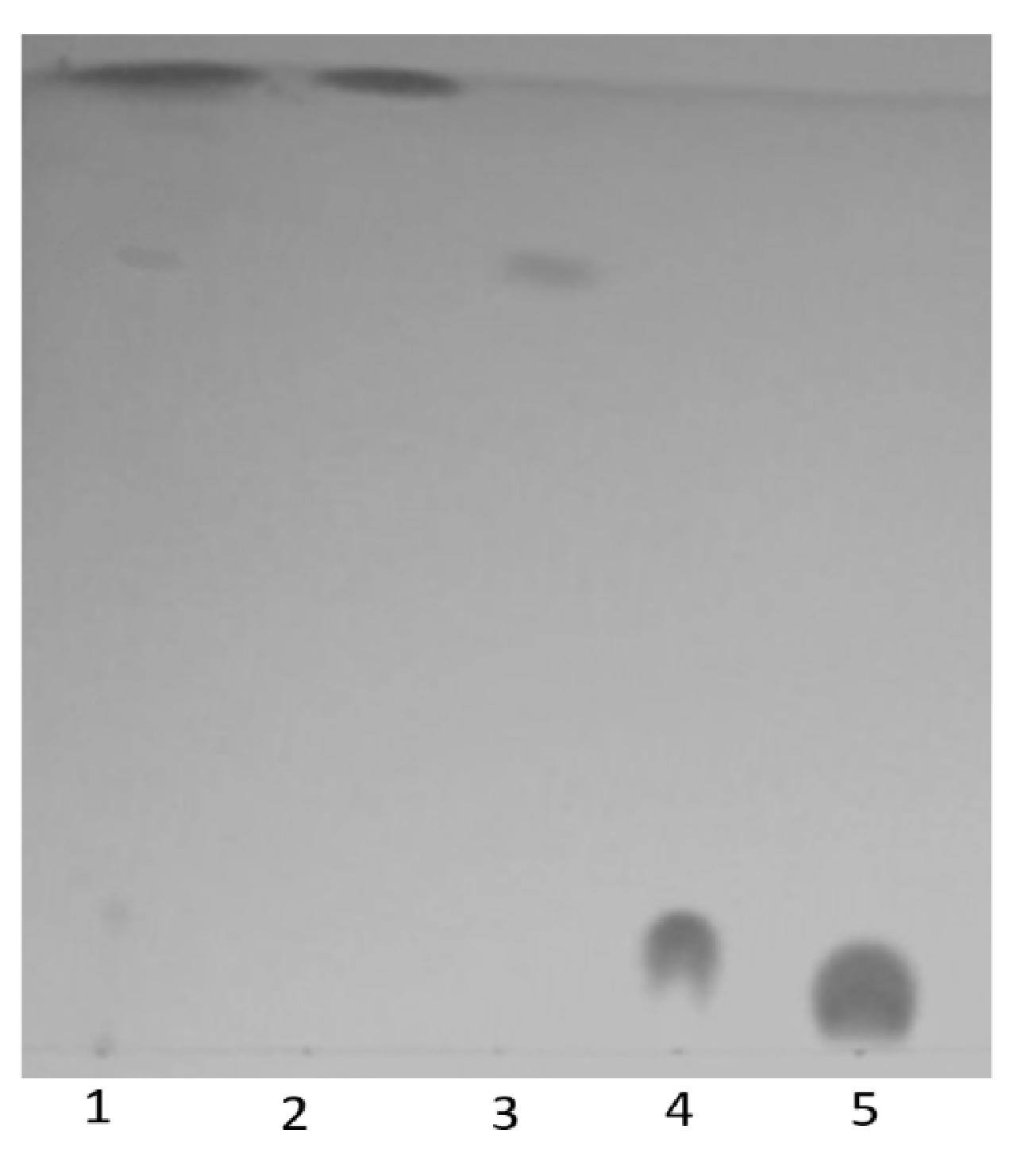

Supplement: Supplementary file 6 — Additional file 6: Figure S5 TLC profile of cell-free extract-mediated transformation of di(2-ethylhexyl) phthalate (DEHP) on silica gel GF254 plate. Lane 1, Organic extract of cell-free extract-catalyzed reaction mixture, incubated for 30 min; lane 2–5, authentic DEHP, mono(2-ethylhexyl) phthalate (MEHP), phthalic acid (PA) and protocatechuic acid (PCA), respectively. [file 12934_2023_2096_MOESM6_ESM.tif]

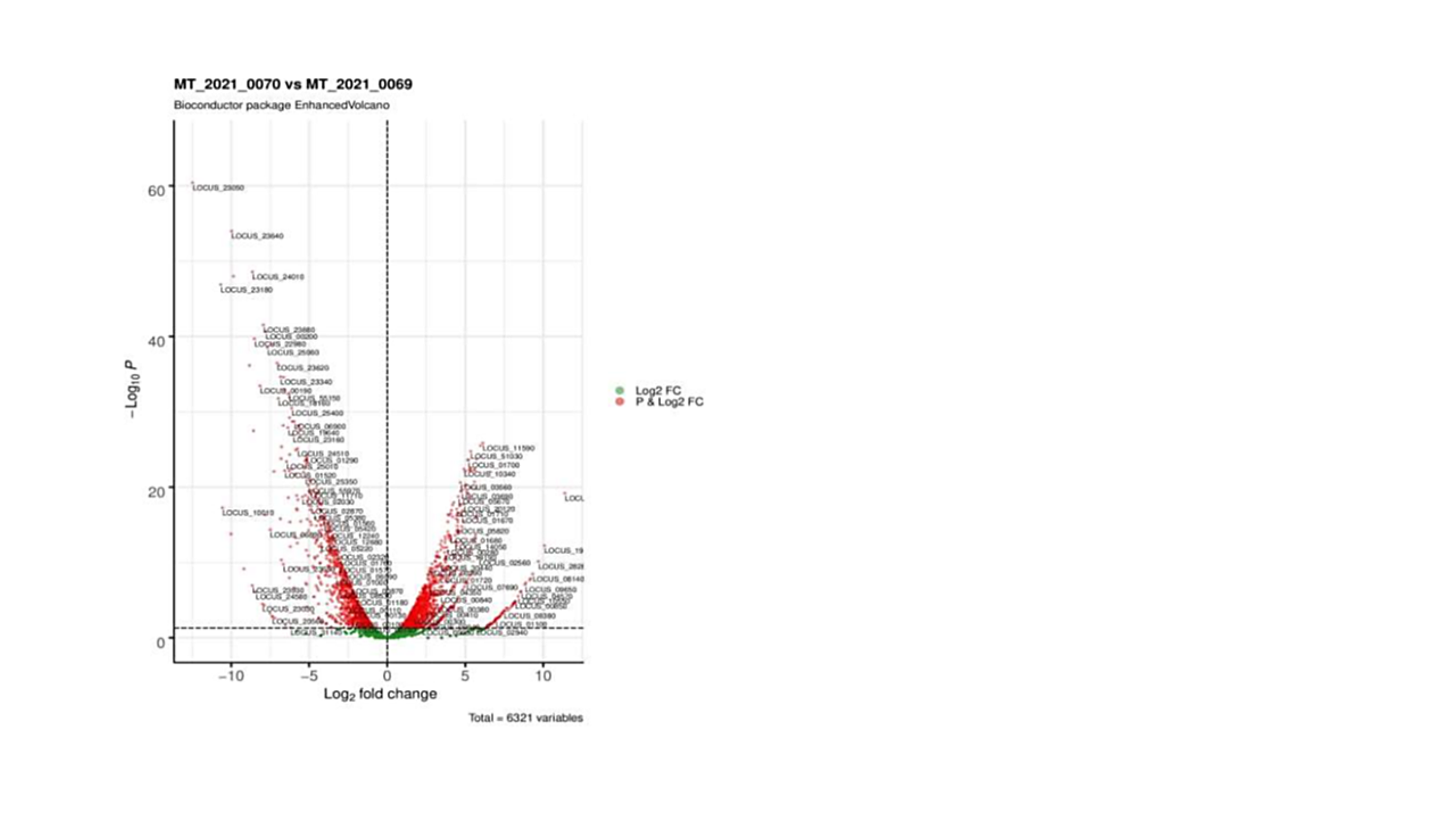

Supplement: Supplementary file 9 — Additional file 9: Figure S6 Volcano plot prepared using the R-library Enhanced Volcano, to represent the differentially expressed genes at p-value cutoff below 0.05, and logFC cutoff value of 0. All the genes marked in red are differentially expressed, while the ones marked in green are not. [file 12934_2023_2096_MOESM9_ESM.tif]

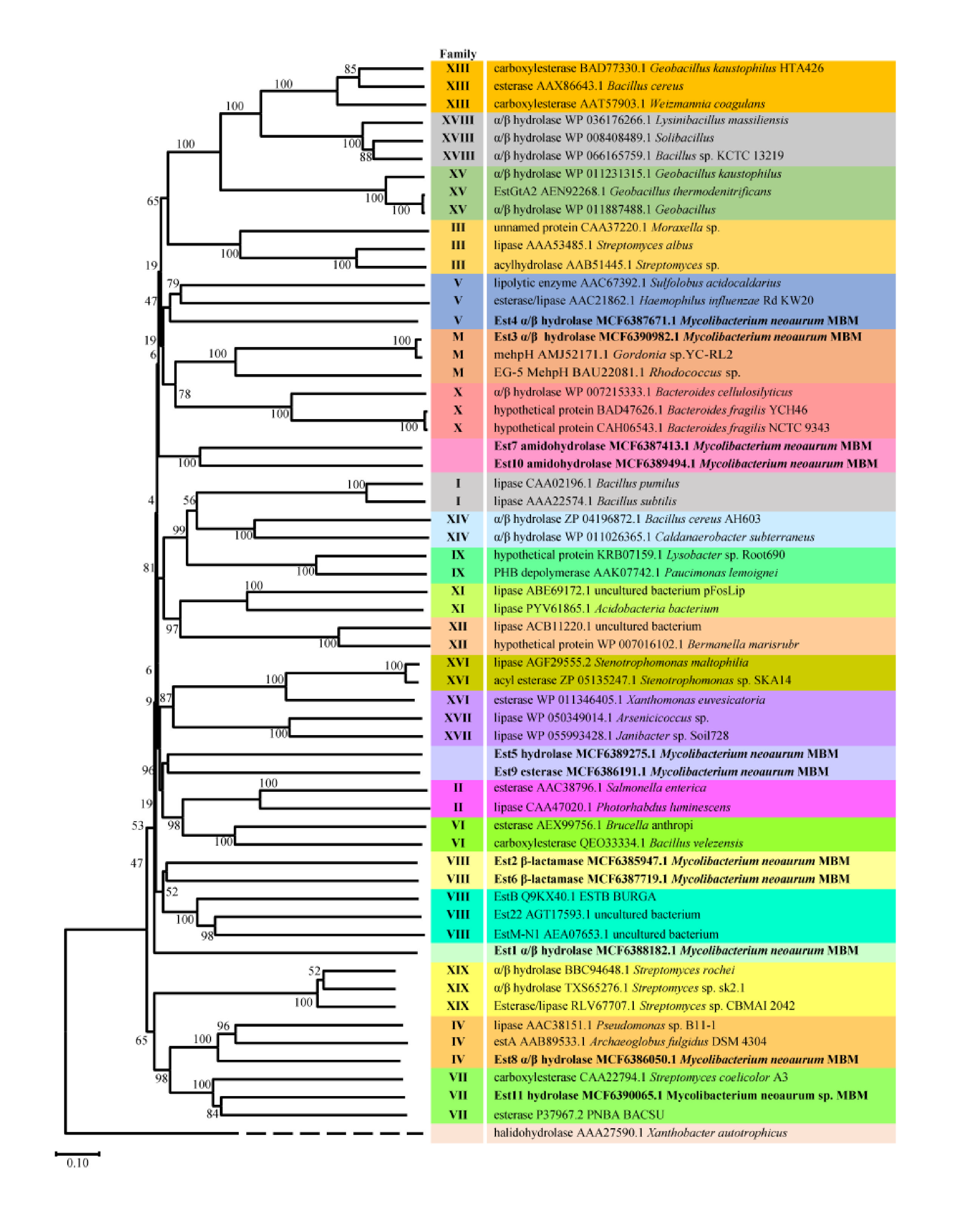

Supplement: Supplementary file 11 — Additional file 11: Figure S7 Phylogenetic analysis of differentially upregulated phthalate hydrolases from strain MBM grown in DEHP-MSM. The upregulated hydrolases are shown in bold letters, while all the enzymes in the tree are mentioned along with respective GenBank accession number and organism name. Numbers at the nodes indicate the levels of bootstrap support based on neighbor joining analysis of 100 resampled data sets. Bootstrap values below 50% are not shown. The scale bar represents 0.10 substitutions per nucleotide position. Multiple sequence alignment was performed using Clustalx v.2 with the inclusion of representative protein sequences from 19 different esterase families. The phylogenetic tree was constructed using neighbour joining algorithm as implemented in Tree ViewX explorer to understand the phylogenetic affiliation of all the upregulated phthalate hydrolases. Halido hydrolase from Xanthobacter autotrophicus was used as an outgroup sequence. [file 12934_2023_2096_MOESM11_ESM.tif]
