# Supplementary material for: Molecular evaluation of the metabolism of estrogenic di(2-ethylhexyl) phthalate in Mycolicibacterium sp
Source: Microb Cell Fact. 2023 Apr 27;22:82. doi: 10.1186/s12934-023-02096-0 (PMC10134610; doi:10.1186/s12934-023-02096-0)
Supplement: Supplementary file 4 — Additional file 4: Table S1 Substrate utilization profile for Mycolicibacterium sp. strain MBM. [file 12934_2023_2096_MOESM4_ESM.docx]

**Additional file 4: Table S1.** Substrate utilization profile for *Mycolicibacterium* sp. strain MBM*^a^*

| **Growth substrate** | **Growth rate (**h^-1^**)** |
| --- | --- |
| Di(2-ethylhexyl) phthalate (DEHP)  Di-*n*-octyl phthalate (D*n*OP)  Dimethyl phthalate (DMP) | 0.305  0.330  0.009 |
| Diethyl phthalate (DEP) | 0.022 |
| Di-*n*-butyl phthalate (D*n*BP) | 0.155 |
| Benzyl butyl phthalate (BBP) | 0.012 |

*^a^*The concentration of each substrate was 0.5 g L^-1^.
