# Supplementary material for: Molecular evaluation of the metabolism of estrogenic di(2-ethylhexyl) phthalate in Mycolicibacterium sp
Source: Microb Cell Fact. 2023 Apr 27;22:82. doi: 10.1186/s12934-023-02096-0 (PMC10134610; doi:10.1186/s12934-023-02096-0)
Supplement: Supplementary file 12 — Additional file 12: Table S5 Primers used for RT-qPCR analysis of selected genes obtained from genome sequence of strain MBM to evaluate genes involved in the degradation of DEHP. [file 12934_2023_2096_MOESM12_ESM.docx]

**Additional file 12: Table S5.** Primers used for RT-qPCR analysis of selected genes obtained from genome sequence of strain MBM to evaluate genes involved in the degradation of DEHP

| Primers | Sequence (5’-3’) |
| --- | --- |
| *est1*_F | GTCGTGAACTACCGGATGAT |
| *est1*_R | ATGTTCTCCGGCGCATAG |
| *est2*_F | ATCACGGTTCGCGATGTATT |
| *est2*_R | GCCATGCGTTCCTCCAT |
| *est3*_F | TTCTCGCGTTGAAGTACCCT |
| *est3*_R | GATGTAGTCCTCGGGCAGAT |
| *est4*_F | GTACCCGCGCAGATCG |
| *est4*_R | CGGATCCGTCGGTTGT |
| *phtAa*_F | CGAACATGAGCTTCGTCCATA |
| *phtAa*_R | AGGACTTCCGTCTCGTTCT |
| *phtAc*_F | TCGGAGCCTCTGACTACTT |
| *phtAc*_R | TACCACCAGTGCGGAGA |
| *pcaG*_F | CCACTACTCGTTCTCGACGAT |
| *pcaG*_R | CAGATATGCGCGTGTGAAGA |
| *pcaL*_F | GCTCACTCATCACGCTGAC |
| *pcaL*_R | GTCTGGAGCAGCAACTCTTT |
| *pcaJ*_F | CCATCTGGACGTGTGTGT |
| *pcaJ*_R | ATTCCGGTCAACGGGTAG |
| *pcaI*_F | GTCCGTAAGGTCATCTGTTC |
| *pcaI*_R | GATCTCGCGCTCTTCTTTG |
| *pcaF*_F | CGTGGAGTCTGGGGATAC |
| *pcaF*_R | AGTTGTTCGTTGCACTGTC |
| *OR1*_F | TGACGATCTGCTCACGAACC |
| *OR1*_R | GATCTGCTGATCGACGGTGA |
| *OR2*_F | TGAAGGTGATGTCGTCGAGC |
| *OR2*_R | CTGCGCGAACCAATTCCATT |
| *OR4*_F | GGCTTCGAGGACCACCTTC |
| *OR4*_R | GGTCAGGGCGTCCACATAG |
| *16s rRNA_*F | GGGTACGAGCCAAGAATCAG |
| *16s rRNA_*R | TCACTCATCGACAACCCTTAAT |
